# Use of CAD/CAM Workflow and Patient-Specific Implants for Maxillary Reconstruction: A Systematic Review

**DOI:** 10.3390/jcm15020647

**Published:** 2026-01-13

**Authors:** Diana D’Alpaos, Giovanni Badiali, Francesco Ceccariglia, Ali Nosrati, Achille Tarsitano

**Affiliations:** 1 Oral and Maxillo-Facial Surgery Unit, IRCCS Azienda Ospedaliero-Universitaria di Bologna, Via Albertoni 15, 40138 Bologna, Italy; giovanni.badiali@unibo.it (G.B.); francesc.ceccarigli2@unibo.it (F.C.); achille.tarsitano2@unibo.it (A.T.); 2 Department of Biomedical and Neuromotor Sciences, Alma Mater Studiorum University of Bologna, 40126 Bologna, Italy; 3 Single Cycle Degree Programme in Medicine and Surgery, Alma Mater Studiorum Università degli Studi di Bologna, 40126 Bologna, Italy; ali.nosrati@studio.unibo.it

**Keywords:** maxilla, reconstruction, surgery, computer-assisted, virtual surgical planning, free flap, patient-specific implant

## Abstract

**Background**: Reconstruction of the maxilla and midface remains one of the most demanding challenges in craniofacial surgery, requiring precise planning and a clear understanding of defect geometry to achieve functional and esthetic restoration. Advances in computer-assisted surgery (CAS) and virtual surgical planning (VSP), based on 3D segmentation of radiologic imaging, have significantly improved the management of maxillary deformities, allowing for further knowledge of patient-specific information, including anatomy, pathology, surgical planning, and reconstructive issues. The integration of computer-aided design and manufacturing (CAD/CAM) and 3D printing has further transformed reconstruction through customized titanium meshes, implants, and surgical guides. **Methods**:This systematic review, conducted following PRISMA 2020 guidelines, synthesizes evidence from clinical studies on CAD/CAM-assisted reconstruction of maxillary and midfacial defects of congenital, acquired, or post-resection origin. It highlights the advantages and drawbacks of maxillary reconstruction with patient-specific implants (PSISs). Primary outcomes are represented by accuracy in VSP reproduction, while secondary outcomes included esthetic results, functions, and assessment of complications. **Results**: Of the 44 identified articles, 10 met inclusion criteria with a time frame from April 2013 to July 2022. The outcomes of 24 treated patients are reported. CAD/CAM-guided techniques seemed to improve osteotomy accuracy, flap contouring, and implant adaptation. **Conclusions**: Although current data support the efficacy and safety of CAD/CAM-based approaches, limitations persist, including high costs, technological dependency, and variable long-term outcome data. This article critically evaluates the role of PSISs in maxillofacial reconstruction and outlines future directions for its standardization and broader adoption in clinical practice.

## 1. Introduction

Reconstruction of the maxilla and midface following oncologic resection or trauma represents one of the most technically complex challenges in craniofacial surgery [1]. Maxillary bones play a key role for the structural and functional keystone of midface, contributing to mastication, speech, swallowing, orbital support, nasal contour, and overall facial esthetics [2]. Defects in this region, especially those resulting from advanced tumor resection, are often extensive and deep, and involve multiple esthetic subunits, whose restoration can result in highly demanding. Some studies demonstrate how quality of life changes and psychological, social, and sexual adjustment of the patients are related to the extent of palatal excision [3]. Maxillary resections turn out to be even more challenging when they are combined with the excision of orbital floor or orbital exenteration [4]. Any reconstructive attempt must simultaneously address the need for oncologic clearance, functional restoration, and satisfying morphological results, other than long-term prosthetic rehabilitation whenever possible [5].

Various autologous and alloplastic options have been developed to reconstruct midfacial defects. Among osseous flaps, the fibular free flap [6] has reported to be the gold standard for large segmental defects due to its reliable vascularity, length, and capacity for simultaneous or delayed implant placement [7,8,9], although its use is limited when restoration of orbitozygomatic projection is needed [10]. Other vascularized bone flaps include the following: iliac crest [11], which has shown satisfactory results in terms of morphological restoration and oral function rehabilitation in numerous cases [12]; osteocutaneous scapular flap, considered by many to be the gold standard for its versatility and shape, providing excellent contour restoration and palatal function [13,14]; and radial forearm flaps, which can be used for soft tissue reconstruction in smaller or anterior defects [15]. While these standard approaches provide structural and functional support, they may lack the precision required for optimal esthetic restoration and implant-supported rehabilitation. Manual intraoperative shaping of flaps and prosthesis meshes can give variability and extended operative time, particularly in large or complex defects, where a free-hand technique can lead to suboptimal results.

In recent years, the introduction of computer-assisted surgery (CAS), more specifically in the form of computer-aided design and manufacturing (CAD/CAM) technologies, has transformed reconstructive strategies in maxillofacial surgery. Relying on high-resolution virtual surgical planning (VSP), CAD/CAM enables surgeons to accurately outline a well-defined and patient-specific plan and allows for the design and fabrication of patient-specific cutting guides and/or implants, titanium meshes, or reconstruction plates whenever needed, and has proven to be a useful tool in complex cases [16,17]. These technologies have proven to be especially beneficial in reconstructing Brown class II–IV maxillary defects, where anatomic complexity is high and spatial accuracy is paramount [18]. CAD/CAM has also facilitated the implementation of immediate implant placement protocols by enabling coordinated planning between the oncologic, reconstructive, and prosthodontic teams [19,20].

Multiple clinical studies now support the use of CAD/CAM in conjunction with various flap types, including the fibula, iliac crest, and soft tissue-only options. Its application spans from the fabrication of precise osteotomy guides to the creation of patient-specific implants that restore midface projection and alveolar ridge continuity. Furthermore, digital workflows allow for pre-operative simulation of prosthetic outcomes for clinical and educational purposes.

This review aims to critically evaluate the current literature on CAD/CAM applications in maxillary and midfacial reconstruction. By synthesizing surgical techniques, outcomes, and reported complications across a diverse set of clinical studies, the present review seeks to define the strengths, limitations, and future directions of CAD/CAM-based reconstruction strategies in complex maxillofacial defects.

## 2. Materials and Methods

This literature review was conducted to evaluate the clinical application and outcomes of CAD/CAM technologies in the context of maxillary resections and subsequent reconstruction. This review aims to synthesize the available evidence from clinical studies that have used CAD/CAM tools, specifically focusing on the manufacturing of patient-specific implants (PSIS) in maxillary reconstruction surgery.

### Search Strategy

This systematic review was conducted in accordance with the PRISMA (preferred reporting items for systematic reviews and meta-analyses) 2020 guidelines. The PRISMA checklist has been compiled and can be found among the Supplementary Materials (Table S1). The protocol for this systematic review was registered in PROSPERO (registration ID: CRD420251242143) prior to data extraction. The protocol outlines the objectives, eligibility criteria, search strategy, and planned methods for study selection, data extraction, and risk-of-bias assessment. Data have been assessed independently by two of the authors, with a process to resolve differences. The risk of bias assessment was performed using Joanna Briggs Institute (JBI)’s critical appraisal tool, more specifically completing the “Checklist for case series” tool for each study selected.

A targeted literature search was conducted independently by two people in different databases, including PubMed (National Library of Medicine, NCBI) and Science Direct, with the time frame being from January 2002 to October 2025. The references of selected articles were also reviewed to identify additional relevant studies. Keywords such as “CAD/CAM”, “maxilla”, “midface”, “virtual surgical planning”, “maxillary reconstruction”, “titanium mesh,” “patient-specific implant,” and “custom-made” were searched alone or in combination, using Boolean operators (AND, OR) to optimize search results.

A total of 1914 records were identified in the first electronic search according to the above-mentioned criteria. Following the initial duplicate exclusion of 35 records, 1808 records were screened by title pertinence, leading to the identification of 71 valid titles. After abstract screening, 44 records were judged as eligible for full-text analysis. A full-text review was performed by two independent reviewers.

**Figure 1:**
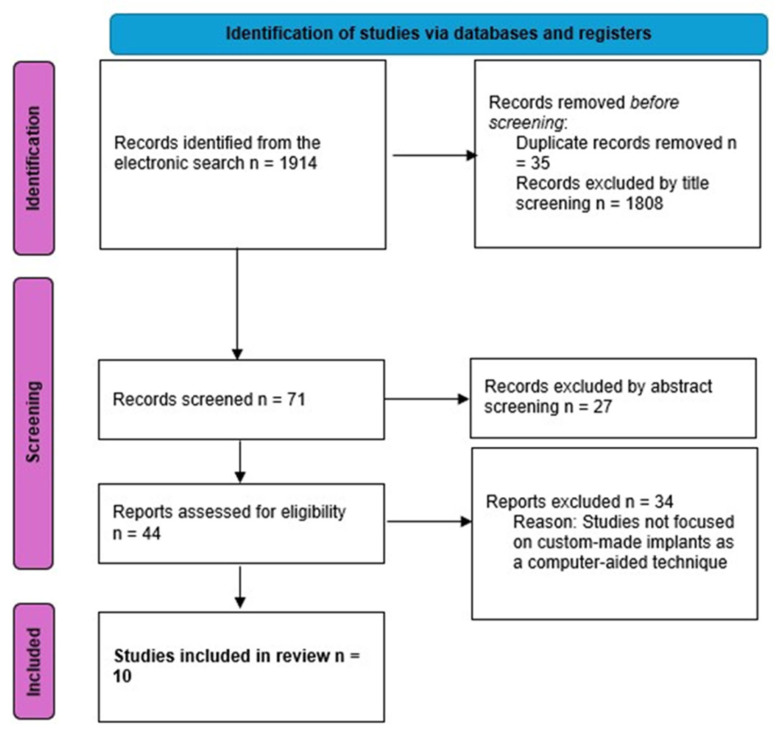
PRISMA workflow for study selection.

Finally, a total of 10 articles were selected based on one or more of the criteria outlined below (Figure 1):

1. Report on clinical application of CAD/CAM technology;
2. Reconstruction following oncologic resection, trauma, or congenital deformity;
3. Use of patient-specific tools and/or devices, such as stereolithographic models, cutting guides and custom plates or prostheses;
4. Accurate description of the use of autologous flaps (e.g., fibula, iliac crest, radial forearm), alone or in combination with alloplastic materials (e.g., titanium implants);
5. Analysis of outcomes related to functional, esthetic, or surgical precision with a clinical and/or radiological follow-up report.

The following exclusion criteria were applied to remove articles that did not focus specifically on the topic:

- Studies reporting non-surgical interventions;
- Reconstruction with CAD/CAM obturator prostheses and/or other prosthodontic rehabilitations;
- Meshes and/or PSIS involving <2 maxillary buttresses: This strict inclusion criterion has been selected in our systematic review to enhance homogeneity among the groups and to focus on more clinically relevant surgical reports of complete maxillary reconstruction;
- Studies focusing on the restoration of a limited area of the maxilla, such as alveolar ridge reconstruction in severely atrophic patients;
- Studies reporting insufficient methodological detail (e.g., lacking information about surgical technique).

From each included study, data were extracted on study design, defect classification (e.g., Brown classification), reconstructive technique, flap or graft type, CAD/CAM elements employed, operative workflow, and reported outcomes. Extracted data were collected in comparative tables to highlight the main features of each selected study, and to underline the potential heterogeneity among them. Since heterogeneity was found among different studies in terms of outcome analysis, it was not possible to proceed to a meta-analysis of the data collected: a narrative exploration of the selected studies has been consequently performed to describe and compare the different characteristics qualitatively.

Specific attention was given to surgical accuracy, implant rehabilitation potential, esthetic restoration, and complication rates.

The included studies were categorized thematically, based on the type of reconstruction performed (e.g., mesh-only vs. microvascular reconstruction), and their reported outcomes were comparatively analyzed with respect to the role and impact of CAD/CAM integration.

- **Primary outcome**: Accuracy in reproduction of VSP through CAD/CAM technique, specifically transferred to the patients through PSIs and principally measured in terms of mean linear distance between reference points in maxillofacial bones from pre-operative to post-operative CT scan.
- **Secondary outcomes**: Esthetic results, functional rehabilitation (in terms of speech, swallowing, and mastication), and assessment of any potential complication; reduction in intraoperative time.

## 3. Results

**Scheme 1:**
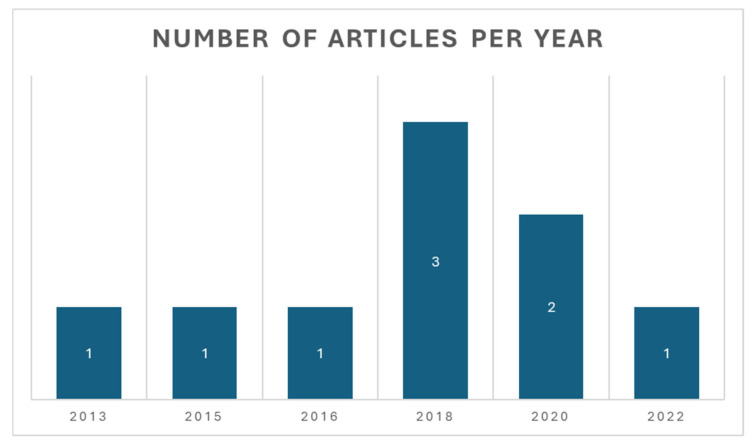
Number of articles published per year.

**Scheme 2:**
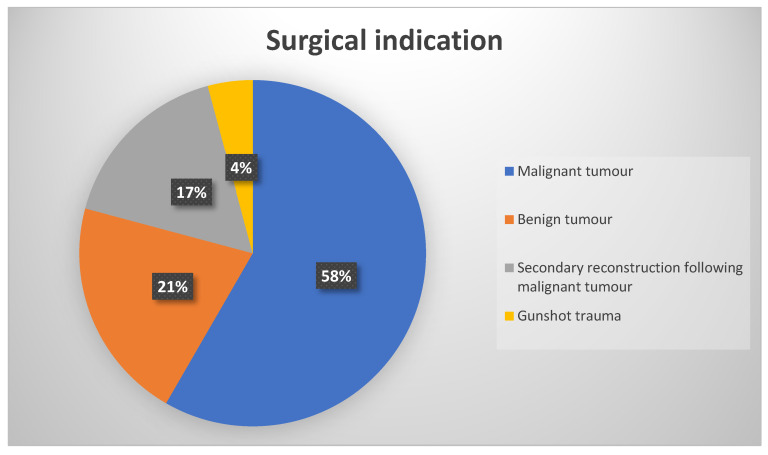
Distribution of studies according to surgical indication.

Of the initial 44 reports assessed for eligibility, a total of 10 peer-reviewed clinical studies published between April 2013 and July 2022 finally met the inclusion criteria for this review (Table 1 and Scheme 1). The 34 excluded reports did not specifically focus on the topic of the present review (i.e., surgical application of VSP through CAD/CAM technique with the use of PSISs in maxillary reconstruction). All selected articles reported on the application of CAD/CAM technologies in the reconstruction of maxillary defects. Most cases involved post-oncologic defects classified as Brown class II–III [18], with reconstruction according to defect type, residual anatomy, and the anticipated prosthetic or functional needs of the patient. A total of 24 treated patients are reported. Details of the pathologies treated with maxillary reconstruction are illustrated in Scheme 2.

A qualitative sensitivity analysis was performed, excluding reports with unclear exposure, absence of properly defined surgical technique, and a poor outcome report or definition.

We identified some concerns in all the studies regarding scientific rigor and transparency. Despite the use of standardized measurement methods (i.e., overlapping of pre- and post-operative CT scans for 3D accuracy), reporting gaps regarding consecutive inclusion of participants, lack of consistency in applying the outcome definitions introduced uncertainty regarding the objectivity of the assessed outcomes. This issue has been addressed by collecting all the data available regarding post-operative outcomes and analyzing them independently in a qualitative manner.

The certainty of evidence for the main outcome (accuracy of VSP application through CAD/CAM technique and PSISs application) was evaluated using the GRADE framework, considering limitations due to risk of bias, inconsistency, imprecision, and publication bias. Certainty was rated as moderate due to the imprecise evaluation of the estimated effect (i.e., improvement of functional and morphological outcomes in patients treated with PSISs vs. patients treated with standard techniques, such as free hand surgery) and to the lack of comparative statistical analysis in the report of results such as multivariate analyses or parametric statistical tests. No serious concerns were identified regarding inconsistency, indirectness, or publication bias. Overall, judgment about risk of bias was considered to be moderate.

### 3.1. CAD/CAM Applications

**Figure 2:**
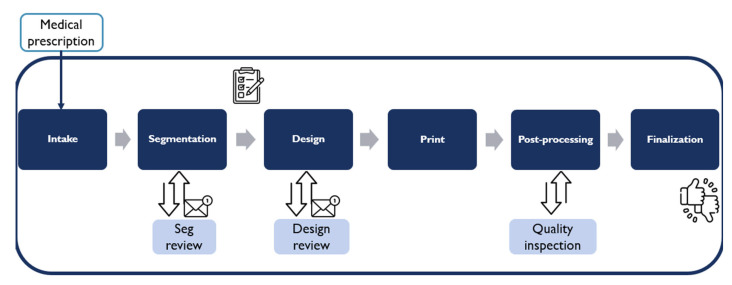
CAD/CAM workflow. Symbol with double arrow linked to envelope represents the communicative workflow between biomedical engineer and surgeon, who gives prompt feedback to proceed or revise. Symbol with list checking represents the VSP with objective assessment of resective and reconstructive aims. Symbol with hands represents final feedback which must be given to biomedical engineer.

All included studies used at CAD/CAM workflow (Figure 2) with the final custom-made manufacturing of a PSIS (mesh or plate), inserted either to support flaps or to restore midface projection. Virtual surgical planning (VSP) was implemented to define the dimension, three-dimensional extension, and morphology of the tumor in oncologic cases; rate and type of alteration for post-oncologic cases; and subsequent osteotomies and bone remodeling plan regarding the flap position and its alignment to maxillary or zygomatic contours when planned. Cutting guides, typically fabricated via 3D printing and composed in acrylic materials, were mostly applied in fibula reconstructions to ensure osteotomy precision and reduce intraoperative variability [23,24,25]. As reported in one of the cases presented by Yang et al. in 2020 [28], the fibular bone can also be segmented, folded, and fixed in alignment with the patient-specific surgical plate.

All the studies utilized outsource software and 3D printers to produce the VSP, surgical guides, and prostheses.

Two authors [22,23] described the use of surgical navigation as an intraoperative aid for monitoring osteotomies and underlying structures, determining the location of the printed titanium mesh, and eventually fixing the fibular flaps.

### 3.2. Reconstructive Strategies, Tissue Sources, and Dental Rehabilitation

A limited range of reconstructive strategies was reported across the selected studies. The fibula free flap was the main reported microvascular flap used for bone reconstruction in large segments or defects requiring restoration of the alveolar ridge. This flap provided sufficient length, adequate thickness, and reliable vascularity. Moreover, it has proven to be effective in re-establishing maxillary projection, carrying out satisfactory morphological results, and contributing to the swallowing function’s rehabilitation. In two cases, one presented by Tarsitano et al. [23] and the other by Yang et al. [28], dental rehabilitation of the reconstructed alveolar ridge was successfully completed with n°4 dental implants positioned in two fibular segments in both cases. In these cases, implant positioning was improved by optimized flap alignment and alveolar ridge positioning, facilitating early prosthetic loading and showing a higher adequacy of fibular free flaps compared with other reconstructive techniques.

In one case, presented in 2013 by Mertens et al. [21], a microvascular osteomyocutaneous scapular flap was used to reconstruct the alveolar ridge of the right maxilla after the resection of a sarcoma. In this case, dental rehabilitation was performed 4 years after surgery because of the loss of the residual teeth due to periodontitis, with n°3 implants positioned in the scapular bone after further bone grafting.

A series presented by Swendseid et al. [27] reports three cases of patients treated with subscapular osteomyocutaneous free flaps, following maxillectomies fixed with custom plates. They present a case of immediate placement of n°4 dental implants in the scapular tip during its harvesting for a case of a benign neoplasm, thus not undergoing post-operative radiotherapy.

### 3.3. Functional, Esthetic, and Operative Outcomes

- **Primary outcome**: Accuracy in the reproduction of VSP through the **CAD/CAM** technique.

Selected studies employing CAD/CAM workflow and PSIS reported high accuracy between pre-operative planning and post-operative skeletal outcomes, based on thepost-operativee CT scan’s comparison with .stl files of pre-operative VSP through an overlapping technique, measured in mm deviation (often within 1–2 mm). Melville et al. [24] present a 3D comparison of pre- and post-operative CT scans, showing 2.2 mm as the greatest discrepancy in their reported case, due to the variability of the positioning of the fibular cutting guide; however, this little discrepancy did not influence the final clinical premaxillary projection. As reported by Tarsitano [23], good reproducibility of pre-operative planning was obtained with less than 1 mm of deviation globally in bone repositioning; good soft tissue repositioning was obtained with an average error of 2.9 mm, particularly located in the cheek area.

Yang et al. [28] reported the following mean absolute distance deviation (including maxilla and mandible cases): 1.5 ± 0.5 mm in the study group and 2.1 ± 0.7 mm in the control group (*p* = 0.003).

In the series presented by Swendseid et al. [27], higher distance values are reported between the planned and actual positioning of the subscapular free segments in anteroposterior, vertical and lateral projection, reaching a mean value of 7.8 mm.

- Secondary outcomes

All the authors reported valid functional results, besides the overall satisfaction of the patients treated in all cases, although no objective assessment was reported except for in one group, in which patient-reported outcomes were explicit: quality of life (QoL) assessment showed no statistical differences in the CAD/CAM group versus the control group (FACT-HN test) [27].

The use of CAD/CAM resulted in reduced overall operative time according to some authors [23,24], while one author declares the possibility of an increased operative time, which could be due to the learning curve necessary for those who are not used to this technology. Yang [28] performed a statistical analysis, which showed no significative difference in operative time between the CAD/CAM group and the control group (*p* = 0.99).

### 3.4. Complications

Among the selected studies, only three articles [21,22,23] clearly report complications in their clinical reports: in 2 patients out of 24, mesh exposure was observed (one after adjuvant RT, in conjunction with the retraction of the lower eyelid providing ectropion, due to the impairment of the soft tissue by RT), which identifies a percentage of about 8.35%.

In one patient, an inflammatory response with soft tissue hyperplasia in the maxillary sinus was observed at the CT scan performed at the time of follow-up [22].

Statistical analysis cannot be drawn over this small sample of patients, so that limited generalizability is not warranted; nevertheless, we have observed that these data represent a low incidence of complications if compared with recent studies about long-term complications in patient-specific plates for maxillary reconstruction [31].

## 4. Discussion

The integration of computer-aided design and manufacturing (CAD/CAM) technologies in craniofacial reconstruction represents a significant advancement in craniofacial surgery [32,33]. While this has been widely documented for mandibular reconstruction over the past 15 years [34,35], the literature about midface and maxillary reconstruction specifically is still sparse.

As demonstrated in this review, CAD/CAM has enabled a shift from conventional, intraoperative decision making to pre-operative virtual surgical planning (VSP), allowing for accurate, efficient, and reproducible reconstructions. This is particularly evident in the application of patient-specific cutting guides and custom-made titanium meshes, which collectively minimize intraoperative uncertainty and improve the spatial alignment of both vascularized bone flaps and implants [23,28].

As previously highlighted in a detailed systematic review by GJC van Baar et al. in 2021 [36], when reporting data about maxillary reconstruction, there is a lack of uniformity in image acquisition, maxillary defect classification, and post-operative evaluation methodologies, which limits the legitimate comparisons of post-operative accuracy results between studies. Nevertheless, general post-operative evaluation showed good results in terms of accuracy in replicating pre-operative VSP.

**Figure 3:**
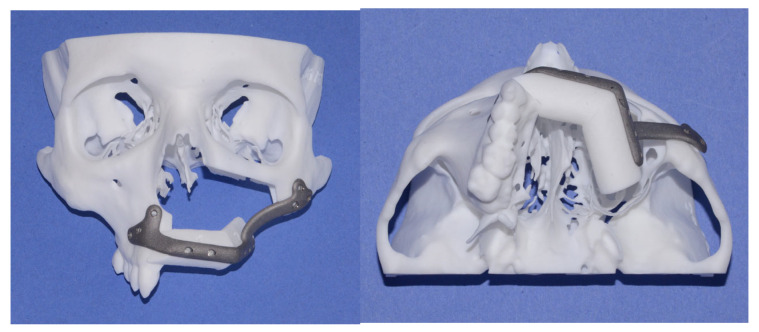
4—Stereolithographic model of post-surgical maxillary defect reconstructed with PSIS and 2 fibular segments correctly positioned, according to VSP.

Among the studies collected in our review, all the authors report satisfying esthetic results at a clinical examination: this appears to be paramount for patients, since it represents the main facial region involved in social interactions and the supporting vital structures, such as the eyes, nose, and upper lip. This could be linked to the high precision of CAD/CAM tools (i.e., cutting guides and/or PSISs) that allow control of the linear and angular positioning of plates or meshes alone or in combination with reconstructive plates (Figure 3). A study made by Al-Sabahi et al. in 2022 demonstrates a lower rate of sagittal and coronal distance between the treated and contralateral side of the face with the use of customized osteotomy guides in patients treated with segmental mandibulectomy [37].

Fibula free flaps, when combined with VSP and cutting guides, continue to serve as the gold standard for large, composite midfacial defects. Their reliable pedicle, sufficient bone length, and compatibility with early implant placement, whenever indicated, make them particularly suited for maxillary reconstruction, providing the chance to restore the alveolar ridge’s height and to perform rehabilitation with dental implants or prostheses [38]. However, success is highly dependent on accurate segmentation, flap modeling, and osteotomy execution steps, which have shown measurable improvement with digital planning tools [39,40].

Notably, custom titanium meshes and patient-specific implants improve esthetic symmetry and reduce intraoperative adjustments [41]. However, their success is closely linked to adequate soft tissue coverage. Cases involving prior radiation or limited flap volume are associated with higher complication rates, including mesh exposure and delayed defect healing [4]. These findings underscore the critical role of flap design and closure techniques, even in digitally planned reconstructions, due to the complex anatomical site and the perceived concerns about it, such as the flap pedicle’s length [1].

In lower-resource settings, studies like the one presented in 2018 by Numajiri et al. [42] have explored in-house CAD/CAM workflows that enable cost-effective surgical planning and guide fabrication without reliance on commercial software. These models demonstrate that digital planning can be adapted to varying economic and institutional conditions while preserving surgical quality. Among the studies selected in our review, none relied upon in-house workflows, since the design and production of titanium implants imply strict quality management and regulatory compliance requirements, including industrial titanium 3D printers, adhering to strict standards for quality and safety.

Despite all the advantages of reconstruction with patient-specific implants, limitations persist. High software and hardware costs, long manufacturing lead times, and the need for multidisciplinary collaboration continue to restrict widespread adoption in some regions. Although the literature evidence about overall costs of CAD/CAM surgery remains scarce, as reported in a study involving a cohort of patients treated for mandibular reconstruction [43], a 115 min saving was observed for the CAD/CAM group compared with the standard group, leading to an overall compensation of the cost of the digital workflow and 3D printing.

Potential disadvantages of 3D-printed implants also include the difficulty in adapting to situations in which the intraoperative surgical plan changes (e.g., positive margins on frozen section examination). For these reasons, the time between virtual planning and surgery must be minimized to avoid any amplification of the tumor margins. CAD/CAM technology, especially that involving PSISs, represents a “rigid” system in surgery: this outlines the essential need for an accurate and targeted VSP that considers potential growth of the tumor in the time lapse between planning and surgery. In cases where rapid growth or uncertain behavior by the tumor is expected, the indication for CAD/CAM technique is excluded.

Another potential disadvantage of the present technique is the learning curve needed to obtain a confident knowledge of the software, although the process of VSP creation usually guarantees a greater knowledge of the surgical case by the medical team involved.

Across included studies, some limitations were observed, including small sample sizes and variability in outcome reporting. About the latter, we have observed an absence of objective assessment of primary and secondary outcomes in all studies. Few studies provided long-term data on implant survival, occlusion function, or quality of life metrics. There remains a lack of consensus on the standardized endpoints for evaluating the success of CAD/CAM reconstructions, which once again hinders metanalysis studies and evidence-based guideline development.

These limitations are probably due to the relatively scarce incidence of cases and the variability of a single case’s features (e.g., amount of tissue loss, quality of tissues around the area to be treated, pre-operative performance status of the patient).

Long-term data on flap stability, implant success, complication rates, and prosthetic integration are essential to establish the durability and cost-effectiveness of CAD/CAM-guided protocols. As technology continues to evolve, future research must emphasize prospective study designs, outcome standardization, and integration with biologically responsive materials to further enhance reconstructive outcomes.

## 5. Conclusions

The lack of uniformity in intraoperative protocols, data collection, and post-operative evaluation of maxillary reconstruction, as well as the variability in reporting post-operative data, put some limits in an objective reliable comparison of the post-operative accuracy results. True evidence of the superiority of CAD-CAM technology integrated with PSISs for maxillary reconstruction cannot be stated. This outlines an overall low level of evidence for the selected studies (IV–V).

Nonetheless, all the authors of the selected studies report satisfying results with the use of PSISs for maxillary reconstruction, confirming small deviations between the post-operative results and VSP.

The integration of CAD/CAM technologies into maxillary and midfacial reconstruction has significantly improved the precision, efficiency, and predictability of complex defect management. Through virtual surgical planning, patient-specific guides, and/or custom implants, surgeons can achieve anatomically accurate reconstructions while reducing intraoperative variability. The fibula free flap remains the most versatile osseous donor site, particularly when combined with CAD-based segmentation and planning tools.

Titanium implants—plates in combination with microvascular flaps or meshes alone or with soft tissue’s support—offer additional consistency, especially in Brown class II–III–IV defects, where two or more maxillary buttresses are lost and anatomical landmarks are no longer identifiable.

The disadvantages of using the CAD/CAM technique in surgical reconstruction of midface and maxillary bones remain the high cost for the whole workflow and the complexity in sourcing the tools needed for some facilities.

Future studies should focus on long-term functional outcomes, cost-effectiveness, and the clinical validation of in-house CAD/CAM solutions. Standardized metrics for evaluating reconstructive success and patient quality of life will be critical in refining protocols and advancing the role of CAD/CAM in contemporary craniofacial surgery.

We believe that future research about CAD/CAM and PSISs application in maxillary reconstruction should be integrated with advanced technologies to guarantee patient-tailored solutions in reconstructive and regenerative surgery.

## Figures and Tables

**Table 1:** Features of the studies selected.

Study (First Author)	Year	Study Design	CAD/CAM Used	Country	Number of Patients	Flap/Material	Defect Type	Complications	Outcomes Analyzed	Ref
**Mertens et al.**	2013	Case report	VSP, cutting guides, custom implant	Germany	1	Scapular free flap, titanium mesh and plate	Brown class III	Mesh exposure (1), ectropion (1)	CT-based accuracy	[21]
**Shan et al.**	2015	Case series	VSP, custom implant, cutting guides	China	2	FFF (1), titanium mesh (2)	Brown class III–V	Soft tissue hyperplasia (1)	CT-based accuracy	[22]
**Tarsitano et al.**	2016	Case series	VSP, cutting guides, custom implants	Italy	4	FFF, titanium meshes and plates	Brown class III	Mesh exposure after RT (1)	CT-based accuracy	[23]
**Melville et al.**	2018	Case report and review	VSP, cutting guides, custom implant	Texas, USA	1	FFF, titanium plate	Brown class IId	None	CT-based accuracy	[24]
**Yang et al.**	2018	Prospective pilot study	VSP, cutting guides, custom plates	China	3	FFF, titanium plates	Brown class III–VI	None	CT-based accuracy	[25]
**Han et al.**	2018	Case report	VSP, custom implants	Korea	3	Polycaprolactone implant, no flap	Brown class II–III	None	CT-based tissue growth	[26]
**Swendseid et al.**	2020	Retrospective study	VSP, cutting guides, custom implants	Pennsylvania, USA	3	Subscapular free flap, titanium plates	Brown class II–V	Not reported	CT-based accuracy	[27]
**Yang et al.**	2020	Prospective study	VSP, cutting guides, custom plates	China	3	FFF (1), not reported (2), titanium plates	Brown class IIId	None	CT-based accuracy	[28]
**Palines et al.**	2021	Case series	VSP, cutting guides, custom plates	Louisiana, USA	3	FFF, titanium plates	Brown class II–III	Not reported	Clinical, radiological	[29]
**Morotomi et al.**	2022	Case series	VSP, custom implant	Japan	1	Titanium mesh, no flap	Brown class III	None	Clinical	[30]

## Data Availability

All data extracted and analyzed during the creation of this systematic review are available from the corresponding author upon reasonable request.

## Supplementary Materials

The following supporting information can be downloaded at: https://www.mdpi.com/article/10.3390/jcm15020647/s1; Table S1: PRISMA checklist.

## Abbreviations

The following abbreviations are used in this manuscript:

CAS	Computer-Assisted Surgery
VSP	Virtual Surgical Planning
CAD/CAM	Computer-Aided Design and Computer-Aided Manufacturing
PSIS	Patient-Specific Implants
